# Efficacy of botanical antifungal and conventional antifungal in the treatment of oral candidiasis: a systematic review and meta-analysis

**DOI:** 10.3389/fphar.2025.1635482

**Published:** 2025-09-25

**Authors:** Nurdiana, Supawadee Naorungroj, Kraisorn Sappayatosok, Luelak Lomlim, Nuntiya Pahumunto

**Affiliations:** ^1^ Candidate of Doctor of Philosophy Program in Oral Health Sciences, Faculty of Dentistry, Prince of Songkla University, Songkhla, Thailand; ^2^ Department of Conservative Dentistry, Faculty of Dentistry, Prince of Songkla University, Songkhla, Thailand; ^3^ Research Center of Excellence for Oral Health, Faculty of Dentistry, Prince of Songkla University, Songkhla, Thailand; ^4^ College of Dental Medicine, Rangsit University, Pathum Thani, Thailand; ^5^ Department of Pharmaceutical Chemistry, Faculty of Pharmaceutical Sciences, Prince of Songkla University, Songkhla, Thailand; ^6^ Phytomedicine and Pharmaceutical Biotechnology Excellence Center, Faculty of Pharmaceutical Sciences, Prince of Songkla University, Songkhla, Thailand; ^7^ Department of Oral Diagnostic Sciences, Faculty of Dentistry, Prince of Songkla University, Songkhla, Thailand

**Keywords:** botanical antifungal, conventional antifungal, alternative treatment, oral candidiasis, clinical outcomes

## Abstract

**Background:**

The use of botanical drugs for treating various disorders has gained increasing attention in recent years, with many studies highlighting the efficacy of botanical antifungals against oral candidiasis. However, there is no definitive evidence indicating whether the botanical antifungals have superior or inferior efficacy compared to the conventional antifungals. This systematic review and meta-analysis evaluated the effectiveness of herbal and botanical antifungals *versus* conventional antifungals in treating oral candidiasis. This is the first pairwise comparison of the clinical efficacy.

**Methods:**

From inception to June 2024, PubMed, EMBASE, Scopus, and Web of Science were searched for randomized clinical trials published in English that investigated botanical antifungals compared to conventional antifungals in treating oral candidiasis. The primary outcome was lesion improvement, with *in vitro Candida* examination as the additional outcome. The lesion improvements were defined as the treatment duration (≤15 days and >15 days). Three independent reviewers screened the papers, and quality was assessed using Cochrane’s Risk of Bias two tools. For the Risk of Bias, five domains were evaluated and classified into three categories: low risk, some concerns, and high risk. A meta-analysis was conducted using STATA version 16 (Texas, United States). The protocol was registered in PROSPERO with an ID of CRD42024589391.

**Results:**

From 1,595 studies identified, 10 trials were included with 426 patients, and 13 botanical drugs were studied. Half (50%) of the included studies had a low risk of bias. Three (30%) studies showed higher efficacy of botanical antifungals, five (50%) studies showed comparable results, and two (20%) studies showed higher efficacy of conventional antifungals in lesion improvement of oral candidiasis. The meta-analysis with random-effects analysis, which encompassed five studies involving 278 patients, revealed no significant difference in lesion improvement for oral candidiasis between botanical and conventional antifungals. The relative risk (RR) was calculated at 0.99, with a 95% confidence interval (CI) of (0.63, 1.56).

**Conclusion:**

Based on the limited evidence, botanical antifungals have comparable efficacy to conventional antifungals in treating oral candidiasis. Therefore, they may serve as adjunctive or alternative treatments.

**Systematic Review Registration:**

https://www.crd.york.ac.uk/PROSPERO/view/CRD42024589391, identifier CRD42024589391.

## 1 Introduction

Oral candidiasis is an opportunistic infection of the oral mucosa caused by an overgrowth of *Candida* strains, influenced by systemic and local factors (Contaldo, 2023). Oral candidiasis is common among the very young and the elderly. About 5%–7% of infants experience oral candidiasis. In patients with AIDS, the prevalence is estimated to range from 9% to 31%, while it is nearly 20% among cancer patients (Patil et al., 2015). Patients with severe oral candidiasis often experience symptoms such as a burning sensation, altered taste, pain, and discomfort that may affect eating ability and subsequently impaired quality of life (Hu et al., 2023).

The current treatment for oral candidiasis includes both topical and systemic antifungal agents (Hu et al., 2023). Topical antifungals are typically recommended for mild cases. In contrast, systemic antifungal therapy is indicated in immunocompromised patients or those at risk of disseminated candidiasis (El-Ansary and El-Ansary, 2023; Hu et al., 2023). Four main categories of antifungal agents are commonly used: polyenes, azoles, echinocandins, and flucytosine (Al Aboody and Mickymaray, 2020; Ordaya et al., 2023). The antifungal that are most frequently utilized are categorized as either polyenes or azoles. The usual dosage for these antifungals is 2–4 times per day for 2–4 weeks. Although the recommended duration of treatment is 2–4 weeks, treatment for oral candidiasis should last at least 2 weeks (Alajbeg et al., 2021).

The global rise in antifungal resistance has escalated the demand for alternative, safe, and effective therapies. Fungi like *Candida* are developing resistance to conventional antifungals (e.g., azoles, polyenes, echinocandins) (Alfadil et al., 2024; Hu et al., 2023). While *Candida albicans* isolates generally remain susceptible to fluconazole, non-*albicans Candida* species exhibit variable susceptibility to antifungal agents (Ordaya et al., 2023). Although nystatin remains one of the most commonly used antifungal agents for topical and oral applications, its prolonged use is limited by concerns related to toxicity, the potential for resistance development, and high recurrence rates. (Anwar et al., 2023). Nystatin, a polyene, is associated with significant toxicity, particularly nephrotoxicity, which limits its clinical use (dos Santos and Branquinha, 2024). Additionally, treatment with nystatin often requires an extended duration of 14–28 days or longer, with occasional adverse effects such as nausea, diarrhea, and loss of appetite (Hu et al., 2023).

Limitations in the efficacy and safety of conventional antifungal agents have led to increased interest in alternative therapeutic strategies that aim to reduce toxicity and improve clinical outcomes. (Contaldo, 2023). While botanical treatments can offer therapeutic benefits, they also come with toxicity risks, especially when misused, at high doses, or alongside conventional medications. Research has explored the potential of discovering new antifungal agents from crude plant extracts, studying botanical medicines like *Psidium guajava* L., *Piper betle* L., *Schefflera leucantha* R. Vig., *Andrographis paniculata* (Burm.f.) Wall. ex Nees, *Garcinia atroviridis* Griff. ex T. Anderson, *Morus alba* L., *Garcinia mangostana* L., *Carthamus tinctorius* L., *Camellia sinensis* (L.) Kuntze, *Aegle marmelos* (L.) Corrêa, and *Rhinacanthus nasutus* (L.) Kurz (Suwanmanee et al., 2014; Jeenkeawpieam et al., 2021).

There are only a few systematic studies directly compare botanical antifungals to conventional antifungals under the same clinical conditions. To address this gap, this study aims to conduct a systematic review and meta-analysis to assess the efficacy of botanical antifungals compared to conventional antifungals for treating oral candidiasis. Importantly, many systematic reviews only evaluate either botanical or conventional antifungals, while this study proposes a pairwise comparison of their efficacy. Additionally, many botanical antifungal reviews are narrative or qualitative in nature; including a meta-analysis brings quantitative strength and objectivity to the discussion. The findings of this research will contribute to a deeper understanding of the role of botanical antifungals in the management of oral candidiasis. Furthermore, the results are expected to provide valuable insights and practical recommendations for integrating diverse botanical antifungal agents into therapeutic protocols for oral candidiasis.

## 2 Materials and methods

This systematic review follows the PRISMA 2020 statement: an updated guideline for reporting systematic review guidelines (Page et al., 2021). The protocol was registered in PROSPERO with an ID of CRD42024589391.

### 2.1 Literature search

Studies eligible for inclusion were randomized clinical trials. Computer searches were conducted electronically for literature published in PubMed, EMBASE, Scopus, and Web of Science published in English until June 2024. The following keywords were used: oral candidiasis AND herbal medicine OR antifungal agents OR therapeutic fungicides AND treatment outcome OR treatment efficacy OR clinical efficacy. In the end, we included ten studies spanning from 2003 to 2023. Supplementary Appendix A presents detailed search terms for each database.

### 2.2 Inclusion and exclusion criteria

Studies were eligible for inclusion in our analysis if they met the following criteria: 1) Randomized clinical trials evaluating the clinical outcomes of botanical antifungals, conventional antifungals, a combination of botanical antifungals, or a combination of botanical and conventional antifungals for the treatment of oral candidiasis; 2) Studies involving human subjects; and 3) Studies published online before June 2024. The exclusion criteria were: 1) Studies published in languages other than English, and 2) Studies without abstracts, those with inaccessible full texts, and duplicates of previously published studies.

### 2.3 Study selection

Three researchers (SN, LL, and N) independently conducted the screening and study selection process. All records were imported into the literature management software EndNote 21 (Clarivate), and duplicate literature was initially removed using this software. Then, the Rayyan software was used to remove other duplicates that EndNote missed. The titles and abstracts of all retrieved literature were pre-screened for potentially eligible studies. Abstracts irrelevant to oral candidiasis, botanical, and conventional antifungal treatments were excluded. Systematic reviews, *in vivo*, *in vitro*, animal studies, protocols, abstracts, comments, and pilot studies were excluded.

Three researchers (SN, KS, and N) used the full text of each study to perform a detailed eligibility assessment. Any disagreements were resolved through consultation, and the final decision was reached through consensus. Studies that did not report clinical outcomes or involve botanical or conventional antifungals were excluded. Improvement in lesions was considered the primary outcome, while *Candida* colony count was included as an additional outcome.

### 2.4 Quality assessment

The risk of bias in each study was assessed using Cochrane’s Risk of Bias two tool for randomized clinical trials. Five domains were evaluated: randomization process, deviations from intended interventions, missing outcome data, measurement of the outcome, and selection of the reported result. Based on this evaluation, the overall risk of bias was classified into three categories: low risk, some concerns, and high risk (Sterne et al., 2019). Three researchers (SN, KS, and N) evaluated these aspects to assess the quality of the included studies. If a study did not provide information on a specific evaluation question, it was classified as “no information” and considered to have some concerns. Any inconsistencies among the researchers regarding the study evaluations were discussed and resolved. Supplementary Appendix B presents a detailed Risk of Bias assessment for each study.

### 2.5 Data extraction

The data extraction included author, publication year, sample size, intervention (botanical antifungal), comparator (conventional antifungal), and clinical outcome (lesion improvement). The results of lesion improvements were grouped according to the treatment duration (≤15 days and >15 days) (Alajbeg et al., 2021). For additional outcomes, the data extraction included author, publication year, intervention (botanical antifungal), comparator (conventional antifungal), and result of *in vitro* examination (*Candida* count). This examination data was categorized as a reduction in the *Candida* colony count. Three researchers (SN, KS, and N) collected the data electronically using Excel sheets.

### 2.6 Data analysis

A meta-analysis was conducted using STATA version 16 (Texas, United States). In the included studies, the researchers used different measurement scales to assess the comparison of lesion improvement on the use of botanical and conventional antifungals in the treatment of oral candidiasis. We used relative risk and 95% confidence intervals (CIs) for binary data, applying a random effect model. Evaluation of heterogeneity (I^2^) was calculated to determine the heterogeneity of the included studies.

## 3 Results

### 3.1 Literature search

**FIGURE 1:**
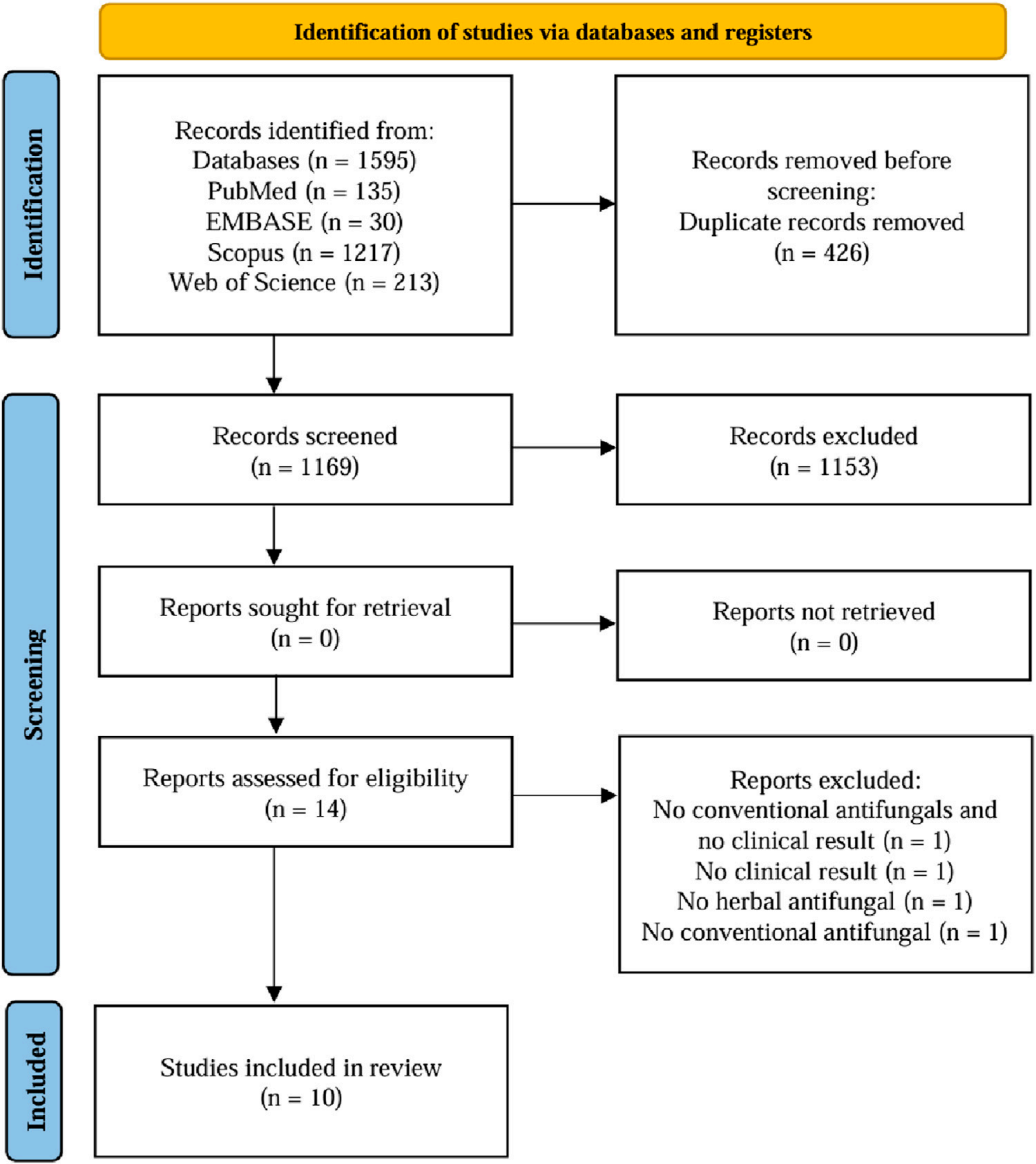
The PRISMA flowchart of the search process for the databases.

A total of 1,595 relevant studies were initially screened, with 135 in PubMed, 30 in EMBASE, 1,217 in Scopus, and 213 in Web of Science. Duplicate records (n = 426) were removed, leaving 1,169 for title and abstract screening. Of those, 1,153 records were excluded based on the inclusion and exclusion criteria. Thus, fourteen studies with full text were assessed for eligibility. Four studies were excluded due to the absence of botanical antifungals, conventional antifungals, or clinical results (Sujanamulk et al., 2020; Santos et al., 2008; Jandourek et al., 1998; Sritrairat et al., 2011) Consequently, ten studies were evaluated for quality of assessment and included in the review (Munkhbat et al., 2023; de Araújo et al., 2021; Tatapudi et al., 2021; Ghorbani et al., 2018; Tay et al., 2014; Pinelli et al., 2013; Bakhshi et al., 2012; Wright et al., 2009; Amanlou et al., 2006; de Souza Vasconcelos et al., 2003). The literature search and selection process were depicted in Figure 1 using a flowchart.

### 3.2 Article characteristics

Table 1 shows the main characteristics of the included articles. All studies included in this review were randomized clinical trials published between 2003 and 2023. The number of subjects in each study ranged from 10 to 30 per group, with participants ranging from under 34–80 years old. Additionally, all studies had a higher number of female subjects. Various types of oral candidiasis were studied, with denture stomatitis being the most common in 80% of the studies.

**TABLE 1:** Main characteristics of the included articles.

Author (Year)	Antifungal	Treatment	Sample Size	Age	Gender (F/M)	Type
Munkhbat et al. (2023)	Botanical	Akhizunber preparation (*Achillea asiatica* Serg. [*Asteraceae*], *Juniperus sabina* L. [*Cupressaceae*], and *Bergenia crassifolia* (L.) Fritsch [*Saxifragaceae*])	25	41–50 years: 12 51–60 years: 10 61–70 years: 22 71–80 years: 6	38/12	Chronic hyperplastic candidiasis Acute pseudomembranous Acute erythematous
	Conventional	Povidone iodine	25			
de Araújo et al. (2021)	Botanical	*Cinnamomum zeylanicum* Blume [Lauraceae]	18	Mean: 57 years	27/9	Denture stomatitis
	Conventional	Nystatin	18			
Tatapudi et al. (2021)	Botanical	*Curcuma longa* L. [*Zingiberaceae*]	25	NI*	13/12	Denture stomatitis
	Conventional	Clotrimazole	25		20/5	
Ghorbani et al. (2018)	Botanical	*Camellia sinensis* (L.) Kuntze [*Theaceae*]	11	Mean: 65 ± 11.3 years	9/2	Denture stomatitis
	Conventional	Nystatin	11		7/4	
Tay et al. (2014)	Botanical	*Uncaria tomentosa* (Willd. ex Schult.) DC. [*Rubiaceae*]	17	Mean: 62.7 years	14/3	Denture stomatitis
	Conventional	Miconazole	15	Mean: 65.8 years	13/2	
Pinelli et al. (2013)	Botanical	*Ricinus communis* L. [*Euphorbiaceae*]	10	Mean: 81.4 ± 9.9 years	24/6	Denture stomatitis
	Conventional	Miconazole Nystatin	Miconazole: 10 Nystatin: 10			
Bakhshi et al. (2012)	Botanical	*Allium sativum* L. [*Amaryllidaceae*]	20	Mean: 73.52 ± 9.81 years	24/1	Denture stomatitis
	Conventional	Nystatin	20			
Wright et al. (2009)	Botanical	*Citrus × limon* (L.) Osbeck [*Rutaceae*] *Cymbopogon citratus* (DC.) Stapf [*Poaceae*]	Lemon juice: 30 Lemon grass: 23	Lemon juice: <34 years, 92.7% Lemon grass: <34 years, 73.4%	Lemon juice: 22/8 Lemon grass: 18/5	Pseudomembranous candidiasis
	Conventional	Gentian violet	29	<34 years, 60.9%	20/9	
Amanlou et al. (2006)	Botanical	*Zataria multiflora* Boiss. [*Lamiaceae*]	12	Mean: 61.9 years	7/5	Denture stomatitis
	Conventional	Miconazole	12	Mean: 59.7 years	7/5	
de Souza Vasconcelos et al. (2003)	Botanical	*Punica granatum* L. [*Lythraceae*]	30	NI*	NI*	Denture stomatitis
	Conventional	Miconazole	30			

NI*: no information.

The botanical antifungals in this review consisted of 13 types of botanical drugs Akhizunber preparation (*Achillea asiatica* Serg., *Juniperus sabina* L., and *Bergenia crassifolia* (L.) Fritsch), *Cinnamomum zeylanicum* Blume, *Curcuma longa* L., *C. sinensis* (L.) Kuntze, *Uncaria tomentosa* (Willd. ex Schult.) DC., *Ricinus communis* L., *Allium sativum* L., *Citrus × limon* (L.) Osbeck, *Cymbopogon citratus* (DC.) Stapf, *Zataria multiflora* Boiss., and *Punica granatum* L. Meanwhile, the conventional antifungals were povidone-iodine, nystatin, clotrimazole, miconazole, and gentian violet. Table 2 provides a summary of the botanical agents used in the studies.

**TABLE 2:** Characteristic of selected botanical agents, their family, and active metabolites.

Scientific Name	Common Name	Family	Active Metabolites
*Achillea asiatica*	Asian yarrow	*Asteraceae*	Flavonoids, terpenoids, lignans, amino acid derivatives, fatty acids, and alkamides
*Juniperus sabina*	Savin juniper	*Cupressaceae*	Sabinene, terpinene 4-ol, myrtenyl acetate, cadinol, and podophyllotoxin
*Bergenia crassifolia*	Heart-leaved bergenia	*Saxifragaceae*	Hamazulene, αpinene, sabinene, limonene, and flavonoids
*Cinnamomum zeylanicum*	Cinnamon	*Lauraceae*	Eugenol, caryophyllene, benzyl benzoate, and linalool
*Curcumin*	Turmeric	*Zingiberaceae*	Polyphenols
*Camellia sinensis*	Tea tree	*Theaceae*	Tannins
*Uncaria tomentosa*	*Cat’s claw*	*Rubiaceae*	Monoterpenoid oxindole alkaloids
*Ricinus communis*	Castor bean	*Euphorbiaceae*	Triglycerides (mainly ricinolein)
*Allium sativum*	Garlic	*Amaryllidaceae*	Allicin, ajoene, diallyl polysulfides, vinyldithiins, S-allyl cysteine, enzymes, saponins, and flavonoids
*Citrus* *limon*	Lemon	*Rutaceae*	Polyphenols, terpenes, and tannins
*Cymbopogon citratus*	Lemon grass	*Poaceae*	Citral, myrcene, citronellal, citronellol, linalool, and geraniol
*Zataria multiflora*	Shirazi thyme	*Lamiaceae*	Thymol and carvacrol
*Punica granatum*	Pomegranate	*Lythraceae*	Sinapyl, flavonoids, coniferyl, anthocyanin, ellagic acid, gallic acid, catechin, ferulic acid, chlorogenic acid, epicatechin, quercetin, rutin, and hydrolyzable tannin

### 3.3 Quality assessment

**FIGURE 2:**
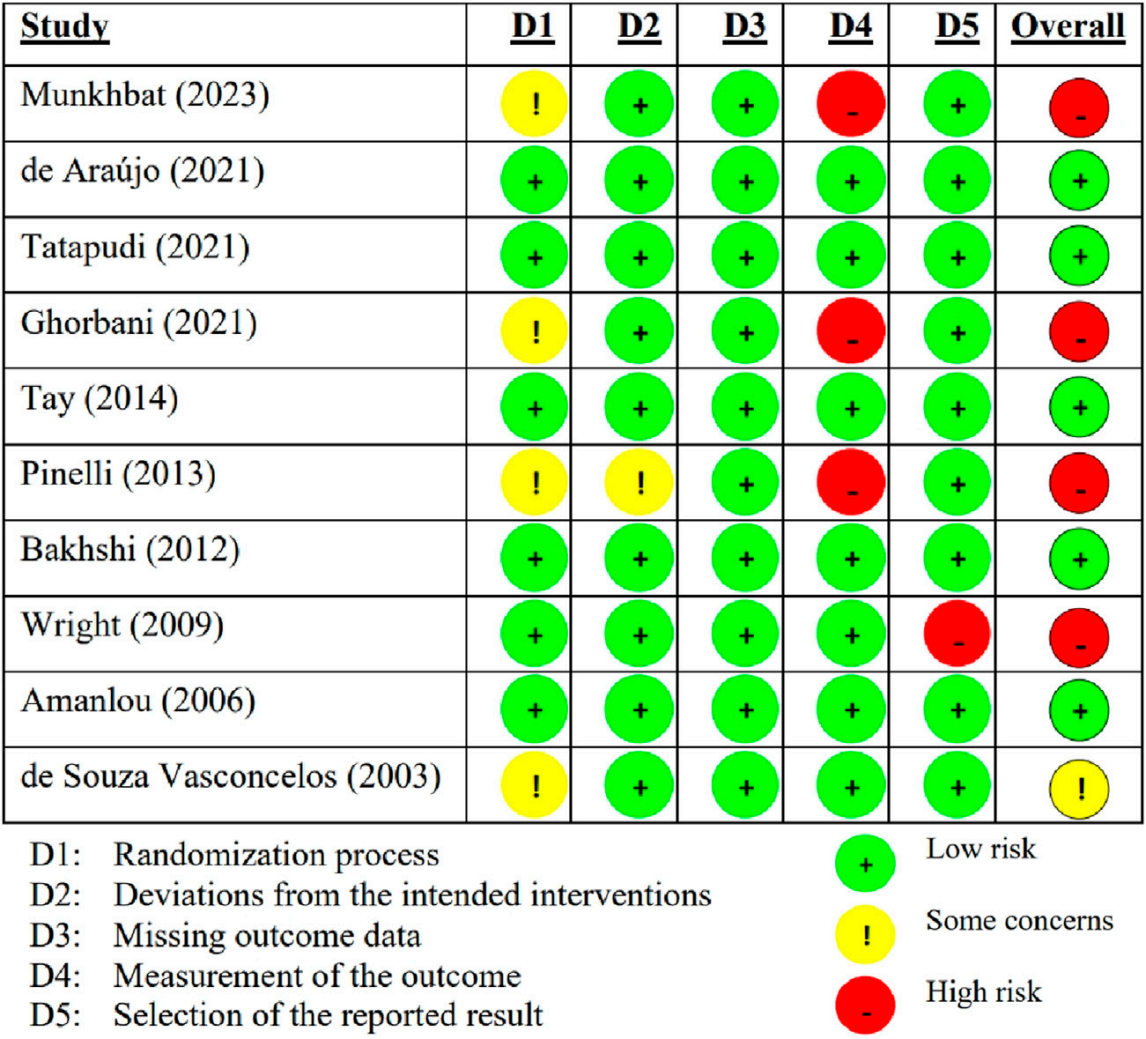
Summary of the risk of bias in the included individual articles according to the RoB 2.0.

**FIGURE 3:**
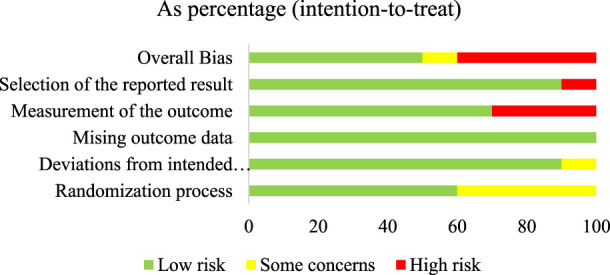
Summary of the risk of bias in all included articles according to the RoB 2.0.

The summary of the risk of bias in the included individual studies according to the RoB 2.0 is shown in Figure 2. Of the ten studies, five (50%) had a low RoB (de Araújo et al., 2021; Tatapudi et al., 2021; Tay et al., 2014; Bakhshi et al., 2012; Amanlou et al., 2006), one (10%) had some concerned RoB (de Souza Vasconcelos et al., 2003), and the other four (40%) had a high RoB (Munkhbat et al., 2023; Ghorbani et al., 2018; Pinelli et al., 2013; Wright et al., 2009). Figure 3 summarizes the risk of bias in all included studies according to RoB 2.0. The measurement of outcomes (Domain 4) raised concern, as three studies, accounting for 30%, exhibited a high RoB. Meanwhile, Domain 1 (randomization process) also raised some concern, with four studies (40%), showed some concerns RoB.

### 3.4 Clinical outcomes

#### 3.4.1 Lesion improvement

Ten studies evaluated the use of botanical and conventional antifungals to treat oral candidiasis. The researchers investigated one preparation that consisted of three botanical drugs and ten individual botanical drugs, for a total of 13 botanical drugs utilized. Three studies showed a higher efficacy of botanical antifungals in lesion improvement than conventional antifungals. The Akhizunber preparation, *C*. *× limon* (L.) Osbeck*, C. citratus* (DC.) Stapf, and *Z. multiflora* Boiss. demonstrated higher efficacy than povidone iodine, gentian violet, and miconazole, respectively (Munkhbat et al., 2023; Wright et al., 2009; Amanlou et al., 2006). Five other studies showed comparable results between botanical and conventional antifungals in treating oral candidiasis. *C*. *zeylanicum* Blume showed a comparable result compared to nystatin, as well as *C. longa* L. to clotrimazole, *C*. *sinensis* (L.) Kuntze to nystatin, *U*. *tomentosa* (Willd. ex Schult.) DC. to miconazole, and *R*. *communis* L. to miconazole and nystatin (de Araújo et al., 2021; Tatapudi et al., 2021; Ghorbani et al., 2018; Tay et al., 2014; Pinelli et al., 2013). In contrast, two studies found conventional antifungals more effective than botanical antifungals in improving lesions. These studies demonstrated that nystatin is more effective than *A*. *sativum* L., and also miconazole against *P*. *granatum* L. (Bakhshi et al., 2012; de Souza Vasconcelos et al., 2003). Table 3 presents the treatment outcomes in terms of lesion improvement.

**TABLE 3:** The treatment outcomes in terms of lesion improvement.

Author (Year)	Type of Treatment	Treatment	Administration Method and Dosage	Lesion Improvement
Munkhbat et al. (2023)	Botanical	Akhizunber preparation	Rinsed and soaked in cotton roll for 20 min for 7 days	≤14 days: 100% >14 days: 0%
	Conventional	Povidone iodine		≤14 days: 100% >14 days: 0%
de Araújo et al. (2021)	Botanical	*C. zeylanicum* Blume	Rinse 10 mL of solution for 1 min and apply the spray to the dentures 3 times/day (every 8 h) for 15 days	Absent; Before: 0%, After: 22% Newton Type I; Before: 61%, After: 56% Newton Type II; Before: 28%, After: 11% Newton Type III; Before: 11%, After: 11%
	Conventional	Nystatin		Absent; Before: 0%, After: 44% Newton Type I; Before: 61%, After: 44% Newton Type II; Before: 39%, After: 11% Newton Type III; Before: 0%, After: 0%
Tatapudi et al. (2021)	Botanical	*Curcumin*	Apply 3 times/day for 28 days	≤14 days: 56% >14 days: 44%
	Conventional	Clotrimazole		≤14 days: 48% >14 days: 52%
Ghorbani et al. (2018)	Botanical	*C. sinensis*	Rinse 15 mL of solution 4 times/day for 14 days	14 days: lesion significantly decreased (P < 0.001)
	Conventional	Nystatin		14 days: lesion significantly decreased (length, P = 0.001) and (width, P = 0.004)
Tay et al. (2014)	Botanical	*U. tomentosa*	Apply 2.5 mL (1 teaspoonful) 3 times/day for 7 days	14 days: the severity diminished over the evaluation periods (7 and 14 days), with no significant differences between the treatments (P > 0.05)
	Conventional	Miconazole		
Pinelli et al. (2013)	Botanical	*R. communis*	Rinse 4 times/day for 30 days	Significant differences between 1st and 30th day (RC – P = 0.011) and 15th and 30th day (RC – P = 0.011)
	Conventional	Miconazole Nystatin	Apply 4 times/day for 30 days	Significant differences between 1st and 30th day (MIC – P = 0.018) and 15th and 30th day (MIC – P = 0.018) No statistically significant differences for the degree of DS in any period (1st vs. 15th day – P = 0.06; 1st vs. 30th day – P = 0.06; 15th vs. 30th day – P = 0.22)
Bakhshi et al. (2012)	Botanical	*A. sativum*	Rinse 20 drops of solution, 3 times/day for 60 s for 28 days	Baseline: width 3.63 ± 1.21 cm, length 3.53 ± 1.16 cm Week 2: width 2.3 ± 0.89 cm, length 2.33 ± 0.92 cm Week 3: width 1.48 ± 0.61 cm, length 1.48 ± 0.67 cm Week 4: width 1.09 ± 0.5 cm, length 0.11 ± 0.21 cm
	Conventional	Nystatin		Baseline: width 3.03 ± 1.03 cm, length 3.61 ± 0.88 cm Week 2: width 1.65 ± 0.79 cm, length 2.08 ± 0.63 cm Week 3: width 0.7 ± 0.43 cm, length 0.79 ± 0.37 cm Week 4: width 0.08 ± 0.18 cm, length 0.99 ± 0.34 cm
Wright et al. (2009)	Botanical	*C. limon* *C. citratus*	*C. limon*: Rinse and spit dilution of 20 mL lemon juice with 10 mL water, then put the mixture in contact with the affected areas as long as possible and swallow. Use 2–3 drops of pure lemon juice 3 times/day for 10 days *C. citratus*: Drink 125 mL of lemon grass infusion 2 times/day for maximum of 10 days	*C. limon*: baseline: scale 1: 6 (20.7%), 2: 13 (44.8%), 3: 9 (31.0%), 4: 2 (6.9%). Clinical success 16, clinical failure 2, withdrawn 12 *C. citratus*: Baseline: scale 1: 7 (24.1%), 2: 6 (20.7%), 3: 9 (31.0%), 4: 1 (3.4%). Clinical success 15, clinical failure 2, withdrawn 6
	Conventional	Gentian violet	Apply 0.5% of the solution 3 times/day for maximum of 10 days	Baseline: scale 1: 9 (31.0%), 2: 12 (41.4%), 3: 6 (20.7%), 4: 2 (6.9%). Clinical success 9, clinical failure 8, withdrawn 12
Amanlou et al. (2006)	Botanical	*Z. multiflora*	Apply 2.5 mL (one teaspoonful) gel to the denture base 4 times/day for 14 days	No significant difference was seen between the two groups (p-values for days 7, 14, 21, and 28 were 0.44, 0.14, 0.59, and 0.75, respectively)
	Conventional	Miconazole		
de Souza Vasconcelos et al. (2003)	Botanical	*P. granatum*	Apply 3 times/day for 15 days	15 days: Satisfactory 7, Regular 14, Unsatisfactory 9
	Conventional	Miconazole		15 days: Satisfactory 19, Regular 8, Unsatisfactory 3

#### 3.4.2 Meta-analysis

**FIGURE 4:**
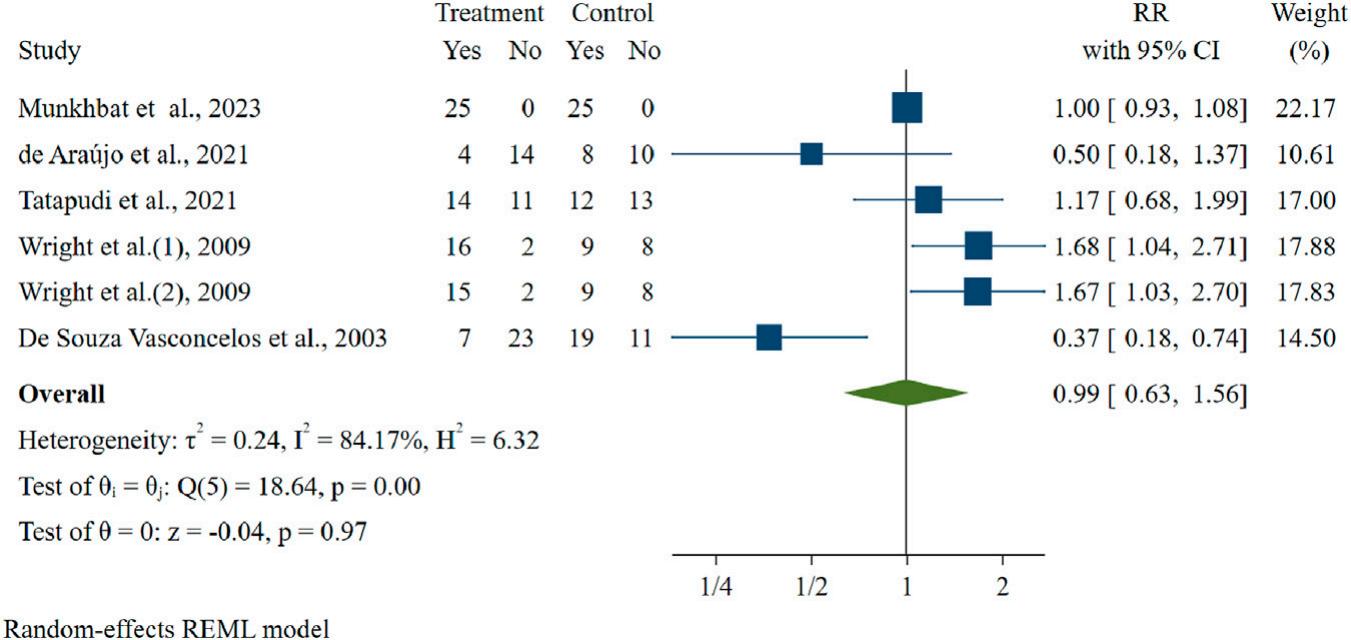
Efficacy of botanical antifungals compared to conventional antifungals in the treatment of oral candidiasis.

A meta-analysis was conducted to compare the efficacy of botanical and conventional antifungals in the treatment of oral candidiasis (Figure 4). Figure 4 presents the meta-analysis results comparing lesion improvement between botanical and conventional antifungals. The efficacy of botanical and conventional antifungals was evaluated in five studies (6 results) (Munkhbat et al., 2023; de Araújo et al., 2021; Tatapudi et al., 2021; Wright et al., 2009; de Souza Vasconcelos et al., 2003). A total of 278 participants were analyzed, and the results showed no significant difference in lesion improvement between botanical and conventional antifungals. The random-effects analysis yielded a relative risk (RR) of 0.99 with a 95% confidence interval (CI) of (0.63, 1.56). However, a high degree of heterogeneity was observed in the analysis, I^2^ = (84.17%). Overall, the meta-analysis suggests that botanical antifungals are as effective as conventional antifungals in improving lesions associated with oral candidiasis.

#### 3.4.3 *Candida* count

Out of the ten studies discussed, eight had results for *Candida* count (Munkhbat et al., 2023; de Araújo et al., 2021; Tatapudi et al., 2021; Ghorbani et al., 2018; Tay et al., 2014; Pinelli et al., 2013; Amanlou et al., 2006; de Souza Vasconcelos et al., 2003), while two studies did not include a laboratory examination (Bakhshi et al., 2012; Wright et al., 2009). Six studies showed comparable results between botanical antifungals and conventional antifungals (Munkhbat et al., 2023; Amanlou et al., 2006; Ghorbani et al., 2018; Pinelli et al., 2013; de Souza Vasconcelos et al., 2003; Tay et al., 2014). In contrast, two studies showed a greater reduction of *Candida* colonies with conventional antifungals in lesion improvement compared to botanical antifungals (de Araújo et al., 2021; Tatapudi et al., 2021). Table 4 presents the *in vitro* results for both treatments.

**TABLE 4:** The treatment outcomes in terms of *Candida count*.

Author (Year)	Type of Treatment	Treatment	Result
Munkhbat et al. (2023)	Botanical	Akhizunber preparation	Akhizunber at concentrations of 1.0%, 2.5%, and 5.0% showed a decreasing effect on *C*. *albicans* biofilm formation
	Conventional	Povidone iodine	
de Araújo et al. (2021)	Botanical	*C. zeylanicum Blume*	*Candida* reduction of 61% (n = 11) of oral mucosa and 33% (n = 6) of dentures
	Conventional	Nystatin	*Candida* reduction of 89% (n = 16) of oral mucosa and 83% (n = 15) of dentures
Tatapudi et al. (2021)	Botanical	*Curcumin*	Mean *Candida* levels, before: 37.080 and after: 3.720
	Conventional	*Clotrimazole*	Mean *Candida* levels, before: 63.960 and after: 14.080
Ghorbani et al. (2018)	Botanical	*C. sinensis*	The mean of *Candida* levels showed significant differences before and after treatment There was no statistically significant difference in the mean of *Candida* levels between groups after the treatment (P = 0.193)
	Conventional	Nystatin	
Tay et al. (2014)	Botanical	*U. tomentosa*	The concentration of colonies was highest on day 0 and diminished in the evaluation periods of day seven and day 14. There were no significant differences between the groups (P > 0.05)
	Conventional	Miconazole	
Pinelli et al. (2013)	Botanical	*R. communis*	No statistically significant difference in intragroup comparisons
	Conventional	Miconazole Nystatin	
Bakhshi et al. (2012)	Botanical	*A. sativum*	-
	Conventional	Nystatin	
Wright et al. (2009)	Botanical	*C. limon* *C. citratus*	-
	Conventional	Gentian violet	
Amanlou et al. (2006)	Botanical	*Z. multiflora*	There were no statistical differences between the groups
	Conventional	Miconazole	
de Souza Vasconcelos et al. (2003)	Botanical	*P. granatum*	After 15 days, positive: 7, and negative: 23
	Conventional	Miconazole	After 15 days, positive: 5, and negative: 25

## 4 Discussion

Oral candidiasis is the most common opportunistic infection of the oral cavity, primarily caused by fungi from the *Candida* genus, particularly *C. albicans* (Alajbeg et al., 2021). The success of oral candidiasis treatment largely depends on administering appropriate antifungals (Sharma, 2019). However, the emerging resistance to antifungal drugs poses serious challenges in managing these infections (Murtaza et al., 2015). Therefore, the development of novel drugs and alternative therapies, including those derived from medicinal plants, has become imperative for the effective treatment of oral candidiasis (Leite et al., 2014). These botanical antifungals offer various mechanisms of action, are biocompatible, and have a lower environmental impact. However, they face challenges in standardization, regulatory approval, clinical validation, and large-scale manufacturing. According to our comprehensive literature search conducted up to June 2024, this systematic review represents the first to undertake a comparative analysis of several botanical and conventional antifungal agents. A total of ten studies meeting the established inclusion criteria were incorporated. The findings indicate that botanical antifungals predominantly demonstrate efficacy that is comparable to or exceeds that of conventional antifungal treatments in the resolution of lesions associated with oral candidiasis. To compare the efficacy of botanical and conventional antifungals, lesion improvements were grouped based on treatment duration (≤15 days and >15 days) to calculate the prevalence of lesion improvement. The treatment durations of ≤15 days and >15 days were determined according to the methods outlined in the reviewed studies, which aligns with clinical practice guidelines for managing oral candidiasis with topical treatments, typically ranging from 7 to 14 days (Quindós et al., 2019).

Overall, the clinical significance is evident in the improvement of lesions treated with botanical antifungals, demonstrating that they can serve as an alternative to conventional antifungal treatments. Three studies demonstrated higher efficacy for botanical antifungals than conventional antifungals. The study by Munkhbat et al. (2023) demonstrated that a mean healing period of 3–5 days was observed in 60% of patients with Akhizunber preparation, while in the Povidone iodine group, all patients recovered in 6–10 days (Munkhbat et al., 2023). The study by Wright et al. (2009) demonstrated that *C*. *× limon* (L.) Osbeck and *C*. *citratus* (DC.) Stapf had better clinical success than gentian violet in treating oral candidiasis in an HIV population (Wright et al., 2009). Furthermore, Amanlou et al. (2006) study indicated that *Z*. *multiflora* Boiss. gel reduced the surface erythema of the palate more efficiently than miconazole (Amanlou et al., 2006).

Five studies demonstrated comparable efficacy in improving lesions associated with oral candidiasis. The study by de Araújo et al. (2021) demonstrated that mouthwash and spray of *C*. *zeylanicum* Blume leaves and nystatin promoted significant clinical improvement of denture-related candidiasis (de Araújo et al., 2021). Tatapudi et al. (2021) study also demonstrated complete resolution of the lesion of denture stomatitis with clotrimazole and *C*. *longa* L. ointment. However, when both groups were analyzed, it was not statistically significant (P = 0.765) (Tatapudi et al., 2021). Moreover, the study by Ghorbani et al. (2018) demonstrated significant decrease in the mean length and width of lesions in the *C*. *sinensis* (L.) Kuntze group (P < 0.001), as well as significant decrease in the mean length (P = 0.001) and width (P = 0.004) of lesions in nystatin group, but no statistically significant difference between the two groups in terms of the mean length (P = 0.179) and width (P = 0.390) of lesions (Ghorbani et al., 2018). Furthermore, the Tay et al. (2014) study demonstrated that the severity of denture stomatitis diminished over the evaluation periods, with no significant differences between *U*. *tomentosa* (Willd. ex Schult.) DC., miconazole, and placebo (P > 0.05) (Tay et al., 2014). Lastly, the study by Pinelli et al. (2013) demonstrated that the efficacy of *R*. *communis* L. was similar to that of the treatment with miconazole (Pinelli et al., 2013).

Meanwhile, two other studies demonstrated that conventional antifungals were more effective at improving lesions in the treatment of oral candidiasis. Bakhshi et al. (2012) found that the reduction in the width of erythema was more pronounced in the nystatin group compared to the *A*. *sativum* L. extract group (Bakhshi et al., 2012). Similarly, de Souza Vasconcelos et al. (2003) reported that miconazole produced better clinical results than *P*. *granatum* L., with this difference being statistically significant (P < 0.01) (de Souza Vasconcelos et al., 2003).

Essential oils are rich sources of phytoactive molecules and are gaining popularity because of their safety, wide potential applications, and significant consumer acceptance (Gupta and Poluri, 2022). Essential oils have extensive biological activity. They are rich mixtures of chemical metabolites belonging to different chemical families, including terpenes, terpenoids, aldehydes, phenols, alcohols, ethers, esters, ketones, and other aromatic and aliphatic constituents with low molecular weights (Gupta and Poluri, 2022; Biernasiuk et al., 2023). Chemical characterization of many essential oils typically reveals that two to three primary metabolites are present in relatively high concentrations (20%–70%), with other elements found in trace amounts (Biernasiuk et al., 2023).

The antimicrobial mechanisms of essential oils are complex and influenced by their chemical composition and the concentration of key individual metabolites (Biernasiuk et al., 2023). In a previous study, essential oils were observed to have anti-*Candida* activity, with the metabolites possibly acting on cell membranes (de Araújo et al., 2021). Some reports revealed that constituents of essential oils mixture can cause cell membrane damage, influence many other cellular activities, including energy production, may be linked to reduced membrane potentials, the disruption of proton pumps, and the depletion of the adenosine triphosphate, the coagulation of cell content, cytoplasm leakage, and finally cell apoptosis or necrosis, leading to cell death (Biernasiuk et al., 2023).

Our meta-analysis demonstrated that botanical antifungals are as effective as conventional antifungals in improving lesions associated with oral candidiasis. This finding is consistent with many studies comparing the efficacy of botanical and conventional antifungals. The diversity of botanical drugs provides a wide range of essential biologically active molecules with enormous potential antifungal properties, such as phenols, tannins, terpenoids, and alkaloids (Hsu et al., 2021). Polyphenols could be classified as phenolic acids, lignin, flavonoids, and tannins (Silva-Beltran et al., 2023).

This study included ten botanical antifungals derived from thirteen different botanical drugs, alongside five conventional antifungal agents. The botanical antifungal included were *Akhizunber* preparation (*A*. *asiatica* Serg., *J*. *sabina* L., and *B*. *crassifolia* (L.) Fritsch), *C*. *zeylanicum* Blume, *C*. *longa* L., *C*. *sinensis* (L.) Kuntze, *U*. *tomentosa* (Willd. ex Schult.) DC., *R*. *communis* L., *A*. *sativum* L., *C*. *× limon* (L.) Osbeck, *C*. *citratus* (DC.) Stapf, *Z*. *multiflora* Boiss., and *P*. *granatum* L. The studies included these botanical drugs because of their biologically active chemical metabolites, such as terpenes, terpenoids, polyphenols (flavonoids and tannins), and alkaloids (de Araújo et al., 2021; Tatapudi et al., 2021; Ghorbani et al., 2018; Tay et al., 2014; Pinelli et al., 2013; Alajbeg et al., 2021; Silva-Beltran et al., 2023; Boncan et al., 2020; Konuk and Ergüden, 2020; Yang et al., 2020; Manso et al., 2022; Shahzad et al., 2014; Dorsaz et al., 2017).

Botanical-derived molecules often target multiple cellular pathways. Terpenes are the most diverse and abundant phytoactive molecules with potent antimicrobial applications. Terpenes are known to modulate ergosterol content in a fungal cell membrane differentially (Gupta and Poluri, 2022). Boncan et al. (2020) also reported that terpenes generate oxidative stress and activate associated cellular response pathways. The increased reactive oxygen species have altered mitochondrial membrane potential, increased Ca^2+^ influx, and cytochrome c flow toward cytosol from mitochondria (Boncan et al., 2020). An optimum level of Ca^2+^ ion is crucial for mitochondrial functioning or ATP production, and any irregularity in its balance leads to apoptosis (Gupta and Poluri, 2022). Terpenoids, the main metabolites of plant volatiles and essential oils, are a large class of natural products exhibiting various biological activities (Konuk and Ergüden, 2020). Terpenoids (isoprenoids) are terpenes containing an oxygen moiety and additional structural rearrangements (Boncan et al., 2020). Raut et al. (2020) found that menthol showed significant biofilm inhibitory activity when studying the effects of plant-derived terpenoids on *C. albicans* (Yang et al., 2020). Phenolic terpenoids disrupt cell membrane integrity and cause leakage of ions, resulting in cell death (Konuk and Ergüden, 2020). Polyphenols are metabolites derived from different parts of plants that contain one or more phenolic groups (Manso et al., 2022). They are macromolecular structures containing phenolic hydroxyl rings (Shahzad et al., 2014). They have many human health benefits, including antioxidant, anti-inflammatory, antibacterial, and antifungal. Studies have reported that plant extracts rich in polyphenols can inhibit the growth of pathogenic fungi (Manso et al., 2022). The polyphenol mechanisms of action could contribute to inhibiting the efflux pump, cell membrane, ergosterol synthesis, and cell wall or produce biofilm damage and reactive oxygen species effect (Silva-Beltran et al., 2023). Shahzad et al. (2014) demonstrated that polyphenols from curcumin and pyrogallol were the most active, inhibiting growth and biofilm formation of *C. albicans* via suppression of genes responsible for adhesion and hyphal growth (Shahzad et al., 2014). Another essential secondary metabolite is alkaloids, which have diverse pharmacological activities. Alkaloids are classified as true alkaloids, protoalkaloids, and pseudoalkaloids. Their mechanism of action is related to membrane permeabilization, inhibition of DNA and RNA, protein synthesis, ergosterol synthesis, and increasing the generation of reactive oxygen species (Silva-Beltran et al., 2023). A study by Dorsaz et al. (2017) showed that the effect of tomatidine isolate of *S. tuberosum* L. alters the regulation of genetics in the ergosterol biosynthesis of *C. albicans*, *C. krusei*, and *Saccharomyces cerevisiae* cells (Dorsaz et al., 2017).

Medicinal plants contain chemical metabolites that may operate singly or combine to heal diseases and improve health (Pammi et al., 2023). Some botanical drugs have various biological activities, including antimicrobial and anti-inflammatory activities, which may be associated with their antioxidant activity (Mahlo et al., 2016). Antioxidants reduce oxidative stress in cells and are, therefore, helpful in treating many human diseases (Pammi et al., 2023). Plants with antioxidant properties mainly contain phenols and flavonoids. Flavonoids play essential roles in preventing diseases associated with oxidative stress (Nwozoa et al., 2023). This metabolite reduces inflammation stress by enhancing the release of systemic mediators, cytokines, and chemokines to induce cellular infiltration, resolve inflammatory responses, and reestablish tissue coordination Gonfa et al., 2023).

Meanwhile, conventional antifungals included povidone iodine, nystatin, clotrimazole, miconazole, and gentian violet (de Souza Vasconcelos et al., 2003; Munkhbat et al., 2023; de Araújo et al., 2021; Tatapudi et al., 2021; Pinelli et al., 2013; Tay et al., 2014; Bakhshi et al., 2012; Wright et al., 2009; Amanlou et al., 2006; Ghorbani et al., 2018) Topical antifungal drugs available include nystatin, miconazole, clotrimazole, and ketoconazole (Taylor et al., 2024). Topical therapy using nystatin and miconazole is still the primary recommended treatment for oral candidiasis due to its high efficacy, low cost, and low side effects (Quindós et al., 2019). Nystatin oral suspension (100000 units/mL) is used in 5 mL orally four times daily (swished for several minutes then swallowed) Taylor et al., 2024). Meanwhile, various topical formulations of miconazole, including buccal tablets, chewing gum, oral gel, and lacquer, have been used to treat oral candidiasis (Zhang et al., 2016). Clotrimazole troches are used at 10 mg orally five times daily (dissolved over 20 min) (Taylor et al., 2024). Both polyenes (nystatin) and azoles (clotrimazole and miconazole) affect fungal plasma membranes by disrupting the synthesis and placement of ergosterol (Contaldo, 2023). Polyenes disrupt ergosterol production, crucial for cell membrane integrity, and can hinder fungal adherence to epithelial cells (Alajbeg et al., 2021). Azoles work by inhibiting the fungal enzyme cytochrome P450 14α-lanosterol demethylase. This leads to the accumulation of toxic methylated intermediates, which disrupt the function of the fungal cell membrane and inhibit its growth (Costa-de-Oliveira and Rodrigues, 2020). Gentian violet is effective against numerous types of pathogenic yeast, such as *Candida*, and has been used in aqueous solutions at 1%–10% concentrations (Kondo et al., 2012). Gentian violet killed *C*. *albicans* at high concentrations but inhibited its virulence by inhibiting adhesion and germ tube production at subinhibitory doses (Hassan et al., 2022). Povidone-iodine is considered to have the broadest spectrum of antimicrobial action, showing efficacy against Gram-positive and Gram-negative bacteria, bacterial spores, fungi, protozoa, and several viruses (Munkhbat et al., 2023). Persistence of effect has also been demonstrated in a study that assessed 1% povidone-iodine as a preprocedural antibacterial agent in individuals with varying degrees of oral hygiene. Povidone-iodine has also shown rapid activity against *Candida in vitro*, ranging between 10 and 120 s from contact to kill time (Kanagalingam et al., 2015).

*Candida* levels were also evaluated as an additional outcome. The results demonstrated that only eight out of 10 studies reviewed also included an *in vitro* examination. Two studies demonstrated higher efficacy for conventional antifungals in reducing the *Candida* levels. These studies used nystatin and clotrimazole as conventional antifungals (de Araújo et al., 2021; Tatapudi et al., 2021). Meanwhile, the other six studies demonstrated comparable results in reducing *Candida* levels using botanical and conventional antifungals (Munkhbat et al., 2023; Amanlou et al., 2006; Ghorbani et al., 2018; Tay et al., 2014; Pinelli et al., 2013; de Souza Vasconcelos et al., 2003). Polyenes are usually fungicidal, and azoles are fungistatic for *Candida* at therapeutic doses. The main mechanisms of antifungal action involve altering the membrane or the fungal cell wall by inhibiting molecules essential for these (Quindós et al., 2019). As mentioned, this mechanism is the same as that of the chemical metabolite in botanical antifungals used to eliminate *Candida*.

To our knowledge, this is the first pairwise comparison of the clinical efficacy that supports the use of botanical antifungals to treat oral candidiasis. This meta-analysis with a random-effect model showed significant heterogeneity (I^2^ = 84.17%), suggesting the need to address this variability with subgroup analysis. Potential sources of heterogeneity in our analysis may arise from several factors related to the studies included. First, we were unable to obtain information about the extraction methods used for the botanical antifungals in the original studies, such as whether they employed aqueous, ethanolic, or other solvent-based extractions. This lack of data could influence the concentration and bioavailability of the active metabolites. Second, there were significant differences in dosages and forms of administration, such as mouth rinses, gels, and lozenges. These variations may impact the local drug concentration and overall treatment efficacy. Finally, the duration of interventions was not standardized across the studies, which might have affected treatment outcomes. These methodological and intervention-related differences could all contribute to the observed heterogeneity. However, the limited number of studies (n = 10) hindered the ability to conduct meaningful subgroup or meta-regression analyses. Further clinical studies should adhere to standardized guidelines for the treatment of oral candidiasis. It is also essential to expand research to include other types of oral candidiasis beyond denture stomatitis. This broader approach will help enhance our understanding and improve treatment options for various forms of oral candidiasis. Additionally, comprehensive studies can provide valuable insights into the efficacy of different antifungal agents and their mechanisms of action in diverse populations.

## 5 Conclusion

In conclusion, this review has thoroughly assessed the efficacy of botanical antifungals for treating oral candidiasis based on clinical trials, providing evidence of their potential as alternative or adjunctive treatments. The results indicate that nearly all botanical antifungals effectively treat oral candidiasis. Due to a specific research question, this study included small sample sizes. Additionally, the significant variation in the botanical antifungals used limits the ability to generate strong clinical recommendations.

## Data Availability

The original contributions presented in the study are included in the article/Supplementary Material, further inquiries can be directed to the corresponding authors.
