# Supplementary material for: Efficacy of botanical antifungal and conventional antifungal in the treatment of oral candidiasis: a systematic review and meta-analysis
Source: Front Pharmacol. 2025 Sep 25;16:1635482. doi: 10.3389/fphar.2025.1635482 (PMC12507744; doi:10.3389/fphar.2025.1635482)
Supplement: Supplementary file 1 [file DataSheet2.pdf]

Unique ID	2023_Akhizunber	Study ID	Munkhbat (2023)	Assessor	N
Ref or Label		Aim	assignment to intervention (the 'intention-to-treat' effect)		
Experimental	Akhizunber Preparation	Comparator	Povidone iodine	Source	Journal article(s)
Outcome	Healing period	Results	Days	Weight	1

Domain	Signalling question	Response	Comments
Bias arising from the randomization process	1.1 Was the allocation sequence random?	NI	
	1.2 Was the allocation sequence concealed until participants were enrolled and assigned to interventions?	NI	
	1.3 Did baseline differences between intervention groups suggest a problem with the randomization process?	N	All diagnosed with oral candidiasis.
	**Risk of bias judgement**	**Some concerns**	Patients were divided into a couple of groups, namely, a treatment group and a control group using a single-blind method. All diagnosed with oral candidiasis.
Bias due to deviations from intended interventions	2.1.Were participants aware of their assigned intervention during the trial?	N	Patients were divided into couple groups, namely, a treatment group and a control group using single-blind method.
	2.2.Were carers and people delivering the interventions aware of participants' assigned intervention during the trial?	NI	
	2.3. If Y/PY/NI to 2.1 or 2.2: Were there deviations from the intended intervention that arose because of the experimental context?	N	
	2.4 If Y/PY to 2.3: Were these deviations likely to have affected the outcome?	NA	
	2.5. If Y/PY/NI to 2.4: Were these deviations from intended intervention balanced between groups?	NA	
	2.6 Was an appropriate analysis used to estimate the effect of assignment to intervention?	Y	The patients were examined, and the primary and secondary morphological elements on the oral mucosa were noticed, the shape, size, and position of the elements were determined
	2.7 If N/PN/NI to 2.6: Was there potential for a substantial impact (on the result) of the failure to analyse participants in the group to which they were randomized?	NA	
	**Risk of bias judgement**	**Low**	
Bias due to missing outcome data	3.1 Were data for this outcome available for all, or nearly all, participants randomized?	Y	
	3.2 If N/PN/NI to 3.1: Is there evidence that result was not biased by missing outcome data?	NA	
	3.3 If N/PN to 3.2: Could missingness in the outcome depend on its true value?	NA	
	3.4 If Y/PY/NI to 3.3: Is it likely that missingness in the outcome depended on its true value?	NA	
	**Risk of bias judgement**	**Low**	
Bias in measurement of the outcome	4.1 Was the method of measuring the outcome inappropriate?	PY	No clear definition for measuring the outcome
	4.2 Could measurement or ascertainment of the outcome have differed between intervention groups?	NI	
	4.3 Were outcome assessors aware of the intervention received by study participants?	NA	
	4.4 If Y/PY/NI to 4.3: Could assessment of the outcome have been influenced by knowledge of intervention received?	NA	
	4.5 If Y/PY/NI to 4.4: Is it likely that assessment of the outcome was influenced by knowledge of intervention received?	NA	
	**Risk of bias judgement**	**High**	
Bias in selection of the reported result	5.1 Were the data that produced this result analysed in accordance with a pre-specified analysis plan that was finalized before unblinded outcome data were available for analysis?	Y	
	5.2 ... multiple eligible outcome measurements (e.g. scales, definitions, time points) within the outcome domain?	N	
	5.3 ... multiple eligible analyses of the data?	N	
	**Risk of bias judgement**	**Low**	
**Overall bias**	**Risk of bias judgement**	**High**	

Unique ID	2021_Cinnamon	Study ID	de Araújo (2021)	Assessor	N
Ref or Label		Aim	assignment to intervention (the 'intention-to-treat' effect)		
Experimental	Cinnamomum zeylanicum Blume	Comparator	Nystatin (100,000 IU/mL)	Source	Journal article(s)
Outcome	Clinical assessment	Results	%	Weight	1

Domain	Signalling question	Response	Comments
Bias arising from the randomization process	1.1 Was the allocation sequence random?	Y	Participants were randomly allocated to treatment and control groups. The allocation sequence was generated with a computer program (random allocation). Allocation concealment was ensured by an individual who was not one of the study investigators to avoid selection bias and the influence of the investigators on the intervention
	1.2 Was the allocation sequence concealed until participants were enrolled and assigned to interventions?	Y	
	1.3 Did baseline differences between intervention groups suggest a problem with the randomization process?	N	
	**Risk of bias judgement**	**Low**	
Bias due to deviations from intended interventions	2.1.Were participants aware of their assigned intervention during the trial?	N	Neither the participants nor the investigators were aware of the allocation groups
	2.2.Were carers and people delivering the interventions aware of participants' assigned intervention during the trial?	N	
	2.3. If Y/PY/NI to 2.1 or 2.2: Were there deviations from the intended intervention that arose because of the experimental context?	NA	
	2.4 If Y/PY to 2.3: Were these deviations likely to have affected the outcome?	NA	
	2.5. If Y/PY/NI to 2.4: Were these deviations from intended intervention balanced between groups?	NA	
	2.6 Was an appropriate analysis used to estimate the effect of assignment to intervention?	Y	
	2.7 If N/PN/NI to 2.6: Was there potential for a substantial impact (on the result) of the failure to analyse participants in the group to which they were randomized?	NA	
	**Risk of bias judgement**	**Low**	
Bias due to missing outcome data	3.1 Were data for this outcome available for all, or nearly all, participants randomized?	Y	Data was available for all participants
	3.2 If N/PN/NI to 3.1: Is there evidence that result was not biased by missing outcome data?	NA	
	3.3 If N/PN to 3.2: Could missingness in the outcome depend on its true value?	NA	
	3.4 If Y/PY/NI to 3.3: Is it likely that missingness in the outcome depended on its true value?	NA	
	**Risk of bias judgement**	**Low**	
Bias in [illegible]	4.1 Was the method of measuring the outcome inappropriate?	N	Data was according to clinical criteria: Type I (pinpoint hyperemia -localized or sparse palatal erythema), Type II (diffuse hyperemia - diffuse erythema, more common), and Type III (granular hyperemia - papillary hyperplasia with rough or nodular mucosa)
	4.2 Could measurement or ascertainment of the outcome have differed between intervention groups?	N	

Domain	Signalling question	Response	Comments
measurement of the outcome	4.3 Were outcome assessors aware of the intervention received by study participants?	N	The investigator responsible for randomization, and the one responsible for preparing the products did not participate in the clinical stage of the study
	4.4 If Y/PY/NI to 4.3: Could assessment of the outcome have been influenced by knowledge of intervention received?	NA	
	4.5 If Y/PY/NI to 4.4: Is it likely that assessment of the outcome was influenced by knowledge of intervention received?	NA	
	**Risk of bias judgement**	**Low**	
**Bias in selection of the reported result**	5.1 Were the data that produced this result analysed in accordance with a pre-specified analysis plan that was finalized before unblinded outcome data were available for analysis?	Y	
	5.2 ... multiple eligible outcome measurements (e.g. scales, definitions, time points) within the outcome domain?	N	
	5.3 ... multiple eligible analyses of the data?	N	
	**Risk of bias judgement**	**Low**	
**Overall bias**	**Risk of bias judgement**	**Low**	

**Unique ID**	2021_Curcumin	**Study ID**	Tatapudi (2021)	**Assessor**	N
**Ref or Label**		**Aim**	assignment to intervention (the 'intention-to-treat' effect)		
**Experimental**	Curcumin	**Comparator**	Clotrimazole	**Source**	Journal article(s)
**Outcome**	Complete resolution of the lesion	**Results**	Weeks	**Weight**	1

Domain	Signalling question	Response	Comments
**Bias arising from the randomization process**	1.1 Was the allocation sequence random?	Y	This was a randomized double-blind clinical trial
	1.2 Was the allocation sequence concealed until participants were enrolled and assigned to interventions?	Y	
	1.3 Did baseline differences between intervention groups suggest a problem with the randomization process?	N	
	**Risk of bias judgement**	**Low**	
**Bias due to deviations from intended interventions**	2.1.Were participants aware of their assigned intervention during the trial?	N	The drug dispensed in similar amber-coloured bottles
	2.2.Were carers and people delivering the interventions aware of participants' assigned intervention during the trial?	NI	
	2.3. If Y/PY/NI to 2.1 or 2.2: Were there deviations from the intended intervention that arose because of the experimental context?	N	
	2.4 If Y/PY to 2.3: Were these deviations likely to have affected the outcome?	NA	
	2.5. If Y/PY/NI to 2.4: Were these deviations from intended intervention balanced between groups?	NA	
	2.6 Was an appropriate analysis used to estimate the effect of assignment to intervention?	Y	
	2.7 If N/PN/NI to 2.6: Was there potential for a substantial impact (on the result) of the failure to analyse participants in the group to which they were randomized?	NA	
	**Risk of bias judgement**	**Low**	
**Bias due to missing outcome data**	3.1 Were data for this outcome available for all, or nearly all, participants randomized?	Y	
	3.2 If N/PN/NI to 3.1: Is there evidence that result was not biased by missing outcome data?	NA	
	3.3 If N/PN to 3.2: Could missingness in the outcome depend on its true value?	NA	
	3.4 If Y/PY/NI to 3.3: Is it likely that missingness in the outcome depended on its true value?	NA	
	**Risk of bias judgement**	**Low**	
**Bias in measurement of the outcome**	4.1 Was the method of measuring the outcome inappropriate?	N	
	4.2 Could measurement or ascertainment of the outcome have differed between intervention groups?	N	
	4.3 Were outcome assessors aware of the intervention received by study participants?	N	
	4.4 If Y/PY/NI to 4.3: Could assessment of the outcome have been influenced by knowledge of intervention received?	NA	
	4.5 If Y/PY/NI to 4.4: Is it likely that assessment of the outcome was influenced by knowledge of intervention received?	NA	
	**Risk of bias judgement**	**Low**	
**Bias in selection of the reported result**	5.1 Were the data that produced this result analysed in accordance with a pre-specified analysis plan that was finalized before unblinded outcome data were available for analysis?	Y	
	5.2 ... multiple eligible outcome measurements (e.g. scales, definitions, time points) within the outcome domain?	N	
	5.3 ... multiple eligible analyses of the data?	N	
	**Risk of bias judgement**	**Low**	
**Overall bias**	**Risk of bias judgement**	**Low**	

**Unique ID**	2018_Camellia sinensis	**Study ID**	Ghorbani (2021)	**Assessor**	N
**Ref or Label**		**Aim**	assignment to intervention (the 'intention-to-treat' effect)		
**Experimental**	Camellia sinensis	**Comparator**	Nystatin	**Source**	Journal article(s)
**Outcome**	The lesion size (length and width)	**Results**	mm	**Weight**	1

Domain	Signalling question	Response	Comments
**Bias arising from the randomization process**	1.1 Was the allocation sequence random?	Y	The patients were randomly assigned into two groups
	1.2 Was the allocation sequence concealed until participants were enrolled and assigned to interventions?	NI	
	1.3 Did baseline differences between intervention groups suggest a problem with the randomization process?	N	This study was conducted on 22 patients with denture stomatitis
	**Risk of bias judgement**	**Some concerns**	
**Bias due to deviations from intended interventions**	2.1.Were participants aware of their assigned intervention during the trial?	NI	
	2.2.Were carers and people delivering the interventions aware of participants' assigned intervention during the trial?	NI	
	2.3. If Y/PY/NI to 2.1 or 2.2: Were there deviations from the intended intervention that arose because of the experimental context?	N	
	2.4 If Y/PY to 2.3: Were these deviations likely to have affected the outcome?	NA	
	2.5. If Y/PY/NI to 2.4: Were these deviations from intended intervention balanced between groups?	NA	
	2.6 Was an appropriate analysis used to estimate the effect of assignment to intervention?	Y	The lesion size (length and width) was measured on the 1st, 7th, and 14th days using a caliper.
	2.7 If N/PN/NI to 2.6: Was there potential for a substantial impact (on the result) of the failure to analyse participants in the group to which they were randomized?	NA	
	**Risk of bias judgement**	**Low**	
**Bias due to missing outcome data**	3.1 Were data for this outcome available for all, or nearly all, participants randomized?	Y	
	3.2 If N/PN/NI to 3.1: Is there evidence that result was not biased by missing outcome data?	NA	
	3.3 If N/PN to 3.2: Could missingness in the outcome depend on its true value?	NA	
	3.4 If Y/PY/NI to 3.3: Is it likely that missingness in the outcome depended on its true value?	NA	
	**Risk of bias judgement**	**Low**	
**Bias in measurement of**	4.1 Was the method of measuring the outcome inappropriate?	N	The lesion size (length and width) was measured on the 1st, 7th, and 14th days using a calliper
	4.2 Could measurement or ascertainment of the outcome have differed between intervention groups?	N	

measurement of the outcome	4.3 Were outcome assessors aware of the intervention received by study participants?	NI	
	4.4 If Y/PY/NI to 4.3: Could assessment of the outcome have been influenced by knowledge of intervention received?	NI	
	4.5 If Y/PY/NI to 4.4: Is it likely that assessment of the outcome was influenced by knowledge of intervention received?	NI	
	**Risk of bias judgement**	**High**	
**Bias in selection of the reported result**	5.1 Were the data that produced this result analysed in accordance with a pre-specified analysis plan that was finalized before unblinded outcome data were available for analysis?	Y	Results show the mean length and width of lesions before and after treatment
	5.2 ... multiple eligible outcome measurements (e.g. scales, definitions, time points) within the outcome domain?	N	
	5.3 ... multiple eligible analyses of the data?	N	
	**Risk of bias judgement**	**Low**	
**Overall bias**	**Risk of bias judgement**	**High**	

**Unique ID**	2014_Uncaria tomentosa	**Study ID**	Tay (2014)	**Assessor**	N
**Ref or Label**		**Aim**	assignment to intervention (the 'intention-to-treat' effect)		
**Experimental**	Uncaria tomentosa	**Comparator**	Miconazole, Placebo	**Source**	Journal article(s)
**Outcome**	Improvement of oral lesion	**Results**	Days	**Weight**	1

**Domain**	**Signalling question**	**Response**	**Comments**
**Bias arising from the randomization process**	1.1 Was the allocation sequence random?	Y	The study was double-blind. According to the stratified randomization list, 50 patients were randomly assigned to 1 of the 3 experimental groups
	1.2 Was the allocation sequence concealed until participants were enrolled and assigned to interventions?	Y	
	1.3 Did baseline differences between intervention groups suggest a problem with the randomization process?	N	
	**Risk of bias judgement**	**Low**	
**Bias due to deviations from intended interventions**	2.1.Were participants aware of their assigned intervention during the trial?	N	The study was double-blind. According to the stratified randomization list, 50 patients were randomly assigned to 1 of the 3 experimental groups
	2.2.Were carers and people delivering the interventions aware of participants' assigned intervention during the trial?	N	
	2.3. If Y/PY/NI to 2.1 or 2.2: Were there deviations from the intended intervention that arose because of the experimental context?	NA	
	2.4 If Y/PY to 2.3: Were these deviations likely to have affected the outcome?	NA	
	2.5. If Y/PY/NI to 2.4: Were these deviations from intended intervention balanced between groups?	NA	
	2.6 Was an appropriate analysis used to estimate the effect of assignment to intervention?	Y	The effectiveness of the treatment was clinically assessed using Newton's criteria
	2.7 If N/PN/NI to 2.6: Was there potential for a substantial impact (on the result) of the failure to analyse participants in the group to which they were randomized?	NA	
	**Risk of bias judgement**	**Low**	
**Bias due to missing outcome data**	3.1 Were data for this outcome available for all, or nearly all, participants randomized?	Y	Of the 50 patients, 2 were withdrawn from the study for not returning to the controls. The remaining 48, who had a mean age of 63.83 +/- 8.9 years, entered the trial
	3.2 If N/PN/NI to 3.1: Is there evidence that result was not biased by missing outcome data?	NA	
	3.3 If N/PN to 3.2: Could missingness in the outcome depend on its true value?	NA	
	3.4 If Y/PY/NI to 3.3: Is it likely that missingness in the outcome depended on its true value?	NA	
	**Risk of bias judgement**	**Low**	
**Bias in measurement of the outcome**	4.1 Was the method of measuring the outcome inappropriate?	N	It was tested on day 0, day 7 (end of treatment), and day 14 (7 days after treatment). At follow-up visits, visual observations were made, and color photographs of the mucosa were taken. The operator was always the same (L.Y.T.), as were the conditions, such as place, light, angle, and patient position
	4.2 Could measurement or ascertainment of the outcome have differed between intervention groups?	N	
	4.3 Were outcome assessors aware of the intervention received by study participants?	N	Two independent calibrated dentists blindly analyzed the photographs taken of each patient
	4.4 If Y/PY/NI to 4.3: Could assessment of the outcome have been influenced by knowledge of intervention received?	NA	
	4.5 If Y/PY/NI to 4.4: Is it likely that assessment of the outcome was influenced by knowledge of intervention received?	NA	
	**Risk of bias judgement**	**Low**	
**Bias in selection of the reported result**	5.1 Were the data that produced this result analysed in accordance with a pre-specified analysis plan that was finalized before unblinded outcome data were available for analysis?	Y	The effectiveness of treatment was clinically assessed using Newton's criteria20 (1962) for scoring the severity of DS, with 0 for a healthy palate.
	5.2 ... multiple eligible outcome measurements (e.g. scales, definitions, time points) within the outcome domain?	N	
	5.3 ... multiple eligible analyses of the data?	N	
	**Risk of bias judgement**	**Low**	
**Overall bias**	**Risk of bias judgement**	**Low**	

**Unique ID**	2013_Ricinus communis	**Study ID**	Pinelli (2013)	**Assessor**	N
**Ref or Label**		**Aim**	assignment to intervention (the 'intention-to-treat' effect)		
**Experimental**	Ricinus communis	**Comparator**	Miconazole, Nystatin	**Source**	Journal article(s)
**Outcome**	Healing improvement	**Results**	days	**Weight**	1

**Domain**	**Signalling question**	**Response**	**Comments**
**Bias arising from the randomization process**	1.1 Was the allocation sequence random?	PY	The elderly were randomly divided into three groups
	1.2 Was the allocation sequence concealed until participants were enrolled and assigned to interventions?	NI	
	1.3 Did baseline differences between intervention groups suggest a problem with the randomization process?	N	At the initial clinical evaluation, no significant difference was found regardless of the degree of oral candidiasis (Newton's classification) among the groups
	**Risk of bias judgement**	**Some concerns**	
**Bias due to**	2.1.Were participants aware of their assigned intervention during the trial?	NI	
	2.2.Were carers and people delivering the interventions aware of participants' assigned intervention during the trial?	NI	
	2.3. If Y/PY/NI to 2.1 or 2.2: Were there deviations from the intended intervention that arose because of the experimental context?	NI	
	2.4 If Y/PY to 2.3: Were these deviations likely to have affected the outcome?	NA	

deviations from intended interventions	2.5. If Y/PY/NI to 2.4: Were these deviations from intended intervention balanced between groups?	NA	
	2.6 Was an appropriate analysis used to estimate the effect of assignment to intervention?	Y	Newton's criteria were used to classify the DS degree
	2.7 If N/PN/NI to 2.6: Was there potential for a substantial impact (on the result) of the failure to analyse participants in the group to which they were randomized?	NA	
	**Risk of bias judgement**	**Some concerns**	
**Bias due to missing outcome data**	3.1 Were data for this outcome available for all, or nearly all, participants randomized?	Y	30 patients were selected to participate, results show 30 patients
	3.2 If N/PN/NI to 3.1: Is there evidence that result was not biased by missing outcome data?	NA	
	3.3 If N/PN to 3.2: Could missingness in the outcome depend on its true value?	NA	
	3.4 If Y/PY/NI to 3.3: Is it likely that missingness in the outcome depended on its true value?	NA	
	**Risk of bias judgement**	**Low**	
**Bias in measurement of the outcome**	4.1 Was the method of measuring the outcome inappropriate?	N	Newton's criteria were used to classify the DS degree
	4.2 Could measurement or ascertainment of the outcome have differed between intervention groups?	N	
	4.3 Were outcome assessors aware of the intervention received by study participants?	NI	
	4.4 If Y/PY/NI to 4.3: Could assessment of the outcome have been influenced by knowledge of intervention received?	NI	
	4.5 If Y/PY/NI to 4.4: Is it likely that assessment of the outcome was influenced by knowledge of intervention received?	NI	
	**Risk of bias judgement**	**High**	
**Bias in selection of the reported result**	5.1 Were the data that produced this result analysed in accordance with a pre-specified analysis plan that was finalized before unblinded outcome data were available for analysis?	Y	
	5.2 ... multiple eligible outcome measurements (e.g. scales, definitions, time points) within the outcome domain?	N	
	5.3 ... multiple eligible analyses of the data?	N	
	**Risk of bias judgement**	**Low**	
**Overall bias**	**Risk of bias judgement**	**High**	

**Unique ID**	2012_Garlic	**Study ID**	Bakhshi (2012)	**Assessor**	N
**Ref or Label**		**Aim**	assignment to intervention (the 'intention-to-treat' effect)		
**Experimental**	Garlic	**Comparator**	Nystatin	**Source**	Journal article(s)
**Outcome**	Healing period	**Results**	Weeks	**Weight**	1

Domain	Signalling question	Response	Comments
**Bias arising from the randomization process**	1.1 Was the allocation sequence random?	Y	This was a randomized double-blind clinical trial.<br>Patients were randomly divided into two treatment groups
	1.2 Was the allocation sequence concealed until participants were enrolled and assigned to interventions?	Y	
	1.3 Did baseline differences between intervention groups suggest a problem with the randomization process?	NI	
	**Risk of bias judgement**	**Low**	
**Bias due to deviations from intended interventions**	2.1.Were participants aware of their assigned intervention during the trial?	N	Garlic aqueous solution or nystatin mouthwash in similar bottles (shape, size and colour)<br>The examiner (an oral medicine specialist) was unaware of the type of mouthwash, and so was the statistician consultant, who was uninformed about the case groups and only knew that there were two study groups marked as A and B
	2.2.Were carers and people delivering the interventions aware of participants' assigned intervention during the trial?	N	
	2.3. If Y/PY/NI to 2.1 or 2.2: Were there deviations from the intended intervention that arose because of the experimental context?	NA	
	2.4 If Y/PY to 2.3: Were these deviations likely to have affected the outcome?	NA	
	2.5. If Y/PY/NI to 2.4: Were these deviations from intended intervention balanced between groups?	NA	
	2.6 Was an appropriate analysis used to estimate the effect of assignment to intervention?	Y	The length and width of erythema underneath the denture were measured in centimeters at different times by an oral medicine specialist using an oral calliper
	2.7 If N/PN/NI to 2.6: Was there potential for a substantial impact (on the result) of the failure to analyse participants in the group to which they were randomized?	NA	
	**Risk of bias judgement**	**Low**	
**Bias due to missing outcome data**	3.1 Were data for this outcome available for all, or nearly all, participants randomized?	Y	
	3.2 If N/PN/NI to 3.1: Is there evidence that result was not biased by missing outcome data?	NA	
	3.3 If N/PN to 3.2: Could missingness in the outcome depend on its true value?	NA	
	3.4 If Y/PY/NI to 3.3: Is it likely that missingness in the outcome depended on its true value?	NA	
	**Risk of bias judgement**	**Low**	
**Bias in measurement of the outcome**	4.1 Was the method of measuring the outcome inappropriate?	N	The length and width of erythema underneath the denture were measured in centimeters at different times
	4.2 Could measurement or ascertainment of the outcome have differed between intervention groups?	N	
	4.3 Were outcome assessors aware of the intervention received by study participants?	N	The examiner (an oral medicine specialist) was unaware of the type of mouthwash, and so was the statistician consultant
	4.4 If Y/PY/NI to 4.3: Could assessment of the outcome have been influenced by knowledge of intervention received?	NA	
	4.5 If Y/PY/NI to 4.4: Is it likely that assessment of the outcome was influenced by knowledge of intervention received?	NA	
	**Risk of bias judgement**	**Low**	
**Bias in selection of the reported result**	5.1 Were the data that produced this result analysed in accordance with a pre-specified analysis plan that was finalized before unblinded outcome data were available for analysis?	Y	
	5.2 ... multiple eligible outcome measurements (e.g. scales, definitions, time points) within the outcome domain?	N	
	5.3 ... multiple eligible analyses of the data?	N	
	**Risk of bias judgement**	**Low**	
**Overall bias**	**Risk of bias judgement**	**Low**	

**Unique ID**	2009_Lemon/Lemon grass	**Study ID**	Wright (2009)	**Assessor**	N
**Ref or Label**		**Aim**	assignment to intervention (the 'intention-to-treat' effect)		
**Experimental**	Lemon/Lemon grass	**Comparator**	Gentian violet	**Source**	Journal article(s)

Outcome	Healing improvement	Results	Scale	Weight	1
**Domain**	**Signalling question**			**Response**	**Comments**
**Bias arising from the randomization process**	1.1 Was the allocation sequence random?			Y	Randomization was done before the start of the study and stored in sealed, opaque, identical envelopes, which were numbered sequentially
	1.2 Was the allocation sequence concealed until participants were enrolled and assigned to interventions?			Y	
	1.3 Did baseline differences between intervention groups suggest a problem with the randomization process?			N	
	**Risk of bias judgement**			**Low**	
**Bias due to deviations from intended interventions**	2.1.Were participants aware of their assigned intervention during the trial?			Y	The study was an open-label study as, once the patient was assigned to a group, the treatment was known to the patient and the registered nurses
	2.2.Were carers and people delivering the interventions aware of participants' assigned intervention during the trial?			Y	
	2.3. If Y/PY/NI to 2.1 or 2.2: Were there deviations from the intended intervention that arose because of the experimental context?			N	
	2.4 If Y/PY to 2.3: Were these deviations likely to have affected the outcome?			NA	
	2.5. If Y/PY/NI to 2.4: Were these deviations from intended intervention balanced between groups?			NA	
	2.6 Was an appropriate analysis used to estimate the effect of assignment to intervention?			Y	The oral thrush was graded according to the oral thrush scale
	2.7 If N/PN/NI to 2.6: Was there potential for a substantial impact (on the result) of the failure to analyse participants in the group to which they were randomized?			NA	
	**Risk of bias judgement**			**Low**	
**Bias due to missing outcome data**	3.1 Were data for this outcome available for all, or nearly all, participants randomized?			Y	Data was available for 82 patients who completed the study of the 90 patients assigned
	3.2 If N/PN/NI to 3.1: Is there evidence that result was not biased by missing outcome data?			NA	
	3.3 If N/PN to 3.2: Could missingness in the outcome depend on its true value?			NA	
	3.4 If Y/PY/NI to 3.3: Is it likely that missingness in the outcome depended on its true value?			NA	
	**Risk of bias judgement**			**Low**	
**Bias in measurement of the outcome**	4.1 Was the method of measuring the outcome inappropriate?			N	The oral thrush graded according to the oral thrush scale
	4.2 Could measurement or ascertainment of the outcome have differed between intervention groups?			N	
	4.3 Were outcome assessors aware of the intervention received by study participants?			Y	The study was an open-label study as, once the patient was assigned to a group, the treatment was known to the patient and the registered nurses
	4.4 If Y/PY/NI to 4.3: Could assessment of the outcome have been influenced by knowledge of intervention received?			N	
	4.5 If Y/PY/NI to 4.4: Is it likely that assessment of the outcome was influenced by knowledge of intervention received?			NA	
	**Risk of bias judgement**			**Low**	
**Bias in selection of the reported result**	5.1 Were the data that produced this result analysed in accordance with a pre-specified analysis plan that was finalized before unblinded outcome data were available for analysis?			N	There's no clear definition for clinical success and clinical failure
	5.2 ... multiple eligible outcome measurements (e.g. scales, definitions, time points) within the outcome domain?			Y	
	5.3 ... multiple eligible analyses of the data?			N	
	**Risk of bias judgement**			**High**	
**Overall bias**	**Risk of bias judgement**			**High**	

Unique ID	2006_Zataria	Study ID	Amanlou (2006)	Assessor	N
Ref or Label		Aim	assignment to intervention (the 'intention-to-treat' effect)		
Experimental	Zataria multiflora	Comparator	Miconazole	Source	Journal article(s)
Outcome	Healing improvement	Results	Scale	Weight	1
**Domain**	**Signalling question**			**Response**	**Comments**
**Bias arising from the randomization process**	1.1 Was the allocation sequence random?			Y	The study was an open, randomized, controlled clinical trial<br>Patients were randomly assigned<br>The identities of the treatment codes were neither known during the examination nor during the statistical analysis.
	1.2 Was the allocation sequence concealed until participants were enrolled and assigned to interventions?			Y	
	1.3 Did baseline differences between intervention groups suggest a problem with the randomization process?			N	All patients with full upper dentures presenting with denture stomatitis defined as confluent erythema and edema of the denture bearing area of palate (type II or III)
	**Risk of bias judgement**			**Low**	
**Bias due to deviations from intended interventions**	2.1.Were participants aware of their assigned intervention during the trial?			N	The identities of the treatment codes were neither known during the examination nor during the statistical analysis.
	2.2.Were carers and people delivering the interventions aware of participants' assigned intervention during the trial?			N	
	2.3. If Y/PY/NI to 2.1 or 2.2: Were there deviations from the intended intervention that arose because of the experimental context?			NA	
	2.4 If Y/PY to 2.3: Were these deviations likely to have affected the outcome?			NA	
	2.5. If Y/PY/NI to 2.4: Were these deviations from intended intervention balanced between groups?			NA	
	2.6 Was an appropriate analysis used to estimate the effect of assignment to intervention?			Y	The erythema of the palatal mucosa was measured with a graded and was recorded according to a 6-point scale
	2.7 If N/PN/NI to 2.6: Was there potential for a substantial impact (on the result) of the failure to analyse participants in the group to which they were randomized?			NA	
	**Risk of bias judgement**			**Low**	
**Bias due to missing outcome data**	3.1 Were data for this outcome available for all, or nearly all, participants randomized?			Y	Twenty-four outpatients were selected, and the results also showed 24 patients
	3.2 If N/PN/NI to 3.1: Is there evidence that result was not biased by missing outcome data?			NA	
	3.3 If N/PN to 3.2: Could missingness in the outcome depend on its true value?			NA	
	3.4 If Y/PY/NI to 3.3: Is it likely that missingness in the outcome depended on its true value?			NA	
	**Risk of bias judgement**			**Low**	
	4.1 Was the method of measuring the outcome inappropriate?			N	
	4.2 Could measurement or ascertainment of the outcome have differed between intervention groups?			N	

Bias in measurement of the outcome	4.3 Were outcome assessors aware of the intervention received by study participants?	N	The study was carried out as a double-blind study
	4.4 If Y/PY/NI to 4.3: Could assessment of the outcome have been influenced by knowledge of intervention received?	NA	
	4.5 If Y/PY/NI to 4.4: Is it likely that assessment of the outcome was influenced by knowledge of intervention received?	NA	
	**Risk of bias judgement**	**Low**	
Bias in selection of the reported result	5.1 Were the data that produced this result analysed in accordance with a pre-specified analysis plan that was finalized before unblinded outcome data were available for analysis?	Y	
	5.2 ... multiple eligible outcome measurements (e.g. scales, definitions, time points) within the outcome domain?	N	
	5.3 ... multiple eligible analyses of the data?	N	
	**Risk of bias judgement**	**Low**	
**Overall bias**	**Risk of bias judgement**	**Low**	

**Unique ID**	2003_Punica granatum	**Study ID**	de Souza Vasconcelos (2003)	**Assessor**	N
**Ref or Label**		**Aim**	assignment to intervention (the 'intention-to-treat' effect)		
**Experimental**	Punica granatum	**Comparator**	Miconazole	**Source**	Journal article(s)
**Outcome**	Satisfactory level	**Results**	Scale	**Weight**	1

Domain	Signalling question	Response	Comments
Bias arising from the randomization process	1.1 Was the allocation sequence random?	Y	The patients were randomly allocated
	1.2 Was the allocation sequence concealed until participants were enrolled and assigned to interventions?	NI	
	1.3 Did baseline differences between intervention groups suggest a problem with the randomization process?	N	
	**Risk of bias judgement**	**Some concerns**	
Bias due to deviations from intended interventions	2.1.Were participants aware of their assigned intervention during the trial?	N	Both gels were stored in 40 g coded tubes
	2.2.Were carers and people delivering the interventions aware of participants' assigned intervention during the trial?	N	
	2.3. If Y/PY/NI to 2.1 or 2.2: Were there deviations from the intended intervention that arose because of the experimental context?	NA	
	2.4 If Y/PY to 2.3: Were these deviations likely to have affected the outcome?	NA	
	2.5. If Y/PY/NI to 2.4: Were these deviations from intended intervention balanced between groups?	NA	
	2.6 Was an appropriate analysis used to estimate the effect of assignment to intervention?	Y	Clinical response was recorded in three levels (satisfactory, regular and unsatisfactory) following the guidelines of Epstein et al.
	2.7 If N/PN/NI to 2.6: Was there potential for a substantial impact (on the result) of the failure to analyse participants in the group to which they were randomized?	NA	
	**Risk of bias judgement**	**Low**	
Bias due to missing outcome data	3.1 Were data for this outcome available for all, or nearly all, participants randomized?	Y	Sixty subjects were selected for the study and results showed 60 subjects
	3.2 If N/PN/NI to 3.1: Is there evidence that result was not biased by missing outcome data?	NA	
	3.3 If N/PN to 3.2: Could missingness in the outcome depend on its true value?	NA	
	3.4 If Y/PY/NI to 3.3: Is it likely that missingness in the outcome depended on its true value?	NA	
	**Risk of bias judgement**	**Low**	
Bias in measurement of the outcome	4.1 Was the method of measuring the outcome inappropriate?	N	Clinical response was recorded in three levels (satisfactory, regular and unsatisfactory) following the guidelines of Epstein et al.
	4.2 Could measurement or ascertainment of the outcome have differed between intervention groups?	N	
	4.3 Were outcome assessors aware of the intervention received by study participants?	N	
	4.4 If Y/PY/NI to 4.3: Could assessment of the outcome have been influenced by knowledge of intervention received?	NA	
	4.5 If Y/PY/NI to 4.4: Is it likely that assessment of the outcome was influenced by knowledge of intervention received?	NA	
	**Risk of bias judgement**	**Low**	
Bias in selection of the reported result	5.1 Were the data that produced this result analysed in accordance with a pre-specified analysis plan that was finalized before unblinded outcome data were available for analysis?	Y	
	5.2 ... multiple eligible outcome measurements (e.g. scales, definitions, time points) within the outcome domain?	N	
	5.3 ... multiple eligible analyses of the data?	N	
	**Risk of bias judgement**	**Low**	
**Overall bias**	**Risk of bias judgement**	**Some concerns**	
