# Supplementary material for: Efficacy of botanical antifungal and conventional antifungal in the treatment of oral candidiasis: a systematic review and meta-analysis
Source: Front Pharmacol. 2025 Sep 25;16:1635482. doi: 10.3389/fphar.2025.1635482 (PMC12507744; doi:10.3389/fphar.2025.1635482)
Supplement: Supplementary file 2 [file DataSheet1.pdf]

Appendix A. Search Methodology

PUBMED search strategy

Component	Step	Searching Syntax	Results
**Domain**	1	oral candidiasis[MeSH Terms]	4,948
	2	oral candidiasis[Text Word]	2,684
	3	oral candidiasis[MeSH Terms] OR oral candidiasis[Text Word]	6,382
**Treatment**	4	herbal medicine[MeSH Terms]	218,933
	5	herbal medicine[Text Word]	18,995
	6	herbal medicine[MeSH Terms] OR herbal medicine[Text Word]	228,383
	7	antifungal agents[MeSH Terms]	69,781
	8	antifungal agents[Text Word]	73,052
	9	antifungal agents[MeSH Terms] OR antifungal agents[Text Word]	73,073
	10	therapeutic fungicides[MeSH Terms]	69,781
	11	therapeutic fungicides[Text Word]	1
	12	therapeutic fungicides[MeSH Terms] OR therapeutic fungicides[Text Word]	69,800
	13	((herbal medicine[MeSH Terms] OR herbal medicine[Text Word]) OR (antifungal agents[MeSH Terms] OR antifungal agents[Text Word])) OR (therapeutic fungicides[MeSH Terms] OR therapeutic fungicides[Text Word])	298,944
**Outcome**	14	treatment outcome[MeSH Terms]	1,291,795
	15	treatment outcome[Text Word]	1,218,568
	16	treatment outcome[MeSH Terms] OR treatment outcome[Text Word]	1,312,411
	17	treatment efficacy[MeSH Terms]	1,291,795
	18	treatment efficacy[Text Word]	22,653
	19	treatment efficacy[MeSH Terms] OR treatment efficacy[Text Word]	1,308,219
	20	clinical efficacy[MeSH Terms]	1,291,795
	21	clinical efficacy[Text Word]	50,674
	22	clinical efficacy[MeSH Terms] OR clinical efficacy[Text Word]	1,328,506
	23	((treatment outcome[MeSH Terms] OR treatment outcome[Text Word]) OR (treatment efficacy[MeSH Terms] OR treatment efficacy[Text Word])) OR (clinical efficacy[MeSH Terms] OR clinical efficacy[Text Word])	1,364,508
	24	((oral candidiasis[MeSH Terms] OR oral candidiasis[Text Word]) AND (((herbal medicine[MeSH Terms] OR herbal medicine[Text Word]) OR (antifungal agents[MeSH Terms] OR antifungal agents[Text Word])) OR (therapeutic fungicides[MeSH Terms] OR therapeutic fungicides[Text Word]))) AND (((treatment outcome[MeSH Terms] OR treatment outcome[Text Word]) OR (treatment efficacy[MeSH Terms] OR	135

		treatment efficacy[Text Word])) OR (clinical efficacy[MeSH Terms] OR clinical efficacy[Text Word]))	

EMBASE search strategy

Component	Step	Searching Syntax	Results
**Domain**	1	oral candidiasis.ab	2713
**Treatment**	2	herbal antifungal*.af	20
	3	antifungal agent*.af	71,305
	4	therapeutic fungicide*.af	8
	5	(herbal antifungal* or antifungal agent* or therapeutic fungicide*).af	71,316
**Outcome**	6	treatment outcome*.af	1,047,727
	7	treatment efficacy.af	34,130
	8	clinical efficacy.af	72,631
	9	(treatment outcome* or treatment efficacy or clinical efficacy).af	1,139,708
	10	(oral candidiasis.ab and (herbal antifungal* or antifungal agent* or therapeutic fungicide*).af and (treatment outcome* or treatment efficacy or clinical efficacy).af)	30

Scopus search strategy

Component	Step	Searching Syntax	Results
Domain	1	TITLE-ABS-KEY (oral AND candidiasis)	14,074
Treatment	2	TITLE-ABS-KEY (herbal AND antifungal)	2,241
	3	TITLE-ABS-KEY (antifungal AND agents)	123,311
	4	TITLE-ABS-KEY (therapeutic AND fungicides)	578
	5	(TITLE-ABS-KEY (herbal AND antifungal)) OR (TITLE-ABS-KEY (antifungal AND agents)) OR (TITLE-ABS-KEY (therapeutic AND fungicides))	127,307
Outcome	6	TITLE-ABS-KEY (treatment AND outcome)	2,550,301
	7	TITLE-ABS-KEY (treatment AND efficacy)	1,139,212
	8	TITLE-ABS-KEY (clinical AND efficacy)	1,128,223
	9	(TITLE-ABS-KEY (treatment AND outcome)) OR (TITLE-ABS-KEY (treatment AND efficacy)) OR (TITLE-ABS-KEY (clinical AND efficacy))	3,557,675
	14	(TITLE-ABS-KEY (oral AND candidiasis)) AND ((TITLE-ABS-KEY (herbal AND antifungal)) OR (TITLE-ABS-KEY (antifungal AND agents)) OR (TITLE-ABS-KEY (therapeutic AND fungicides))) AND ((TITLE-ABS-KEY (treatment AND outcome)) OR (TITLE-ABS-KEY (treatment AND efficacy)) OR (TITLE-ABS-KEY (clinical AND efficacy)))	1,217

Web of Science search strategy

Component	Step	Searching Syntax	Results
**Domain**		ALL=(oral candidiasis)	4,410
**Treatment**		ALL=(herbal antifungal*)	1,366
		ALL=(antifungal agent)	33,151

		ALL=(therapeutic fungicide)	219
		ALL=(herbal antifungal*) OR ALL=(antifungal agent) OR ALL=(therapeutic fungicide)	34,277
**Outcome**		ALL=(treatment outcome)	970,463
		ALL=(treatment efficacy)	641,792
		ALL=(clinical efficacy)	610,717
		ALL=(treatment outcome) OR ALL=(treatment efficacy) OR ALL=(clinical efficacy)	1,681,462
		ALL=(oral candidiasis) AND (ALL=(herbal antifungal*) OR ALL=(antifungal agent) OR ALL=(therapeutic fungicide)) AND (ALL=(treatment outcome) OR ALL=(treatment efficacy) OR ALL=(clinical efficacy))	213

Summary of Search Strategy

**Key concepts**	**Search terms**	**N**
PubMed search	*((oral candidiasis[MeSH Terms] OR oral candidiasis[Text Word]) AND (((herbal medicine[MeSH Terms] OR herbal medicine[Text Word]) OR (antifungal agents[MeSH Terms] OR antifungal agents[Text Word])) OR (therapeutic fungicides[MeSH Terms] OR therapeutic fungicides[Text Word]))) AND (((treatment outcome[MeSH Terms] OR treatment outcome[Text Word]) OR (treatment efficacy[MeSH Terms] OR treatment efficacy[Text Word])) OR (clinical efficacy[MeSH Terms] OR clinical efficacy[Text Word]))*	135
EMBASE search	(oral candidiasis.ab and (herbal antifungal* or antifungal agent* or therapeutic fungicide*).af and (treatment outcome* or treatment efficacy or clinical efficacy).af)	30
Scopus search	(TITLE-ABS-KEY (oral AND candidiasis)) AND ((TITLE-ABS-KEY (herbal AND antifungal)) OR (TITLE-ABS-KEY (antifungal AND agents)) OR (TITLE-ABS-KEY (therapeutic AND fungicides))) AND ((TITLE-ABS-KEY (treatment AND outcome)) OR (TITLE-ABS-KEY (treatment AND efficacy)) OR (TITLE-ABS-KEY (clinical AND efficacy)))	1,217
Web of Science	#13 AND #11 AND #12	213
	Total	1,595
